# Development of the Flexibility in Daily Life scale to measure multidimensional cognitive and behavioural flexibility in health and disease

**DOI:** 10.1111/bjc.12505

**Published:** 2024-09-22

**Authors:** Kristina Horne, Tao Chen, Muireann Irish

**Affiliations:** ^1^ Brain and Mind Centre The University of Sydney Sydney New South Wales Australia; ^2^ School of Psychology The University of Sydney Sydney New South Wales Australia

**Keywords:** behavioural flexibility, cognitive flexibility, compulsivity, executive function, rigidity

## Abstract

**Objectives:**

Inflexibility of thought and behaviour is a transdiagnostic feature of many neuropsychiatric disorders and presents several empirical measurement challenges. Here, we developed and validated the Flexibility in Daily Life scale (FIDL); a novel, self‐report questionnaire, which captures expressions of cognitive and behavioural flexibility in daily life and is sensitive to natural shifts in these processes across the adult lifespan.

**Methods:**

The FIDL was developed using a deductive scale development approach, which aimed to capture common themes within the flexibility literature and across diagnoses (e.g. insistence on sameness, preference for routines). Following multidisciplinary consensus, an initial 37‐item questionnaire was submitted for validation in an online sample of 295 healthy adult participants (19–78 years).

**Results:**

Exploratory factor analysis produced a revised 21‐item version comprising five factors, labelled: *Repetition*, *Switching*, *Predictability/Control*, *Routine*, and *Thoughts/Beliefs*. Internal consistency reliability was good‐to‐strong for the total FIDL score and moderate‐to‐strong for individual subscales. Convergent validity was established between the FIDL and an existing measure of cognitive flexibility. Critically, the FIDL total score evinced a U‐shaped relationship with age, whereby flexibility was lower at the younger and older tails of the lifespan and greater in middle age. The same U‐shaped trajectory emerged for the *Repetition*, *Routine*, and *Thoughts/Beliefs* factors.

**Conclusions:**

Overall, the FIDL is a valid and reliable multidimensional measure of flexibility, which upholds a clearly defined factor structure and good psychometric properties. It promises to be a valuable clinical and research tool to assess the natural fluctuations in flexibility across the lifespan and departures thereof.

**Practitioner points**

- We developed the Flexibility in Daily Life scale (FIDL) to capture natural expressions of cognitive and behavioural flexibility in daily life. The scale measures the capacity to switch between task sets or adopt alternative viewpoints and to flexibly engage in a diverse range of activities and behaviours in daily life.
- The FIDL was validated in healthy adults (19–78 years) and has a clearly defined factor structure, with robust psychometric properties. Five factors emerged, capturing repetitive and stereotypical behaviours (Repetition), the ability to adapt one's task or mental set (Switching), the preference for structured and ordered environments or social settings (Predictability/Control), the preference for routine (Routine), and rigid thinking styles or beliefs (Thoughts/Beliefs).
- Importantly, we demonstrated that the FIDL is sensitive to natural shifts in flexibility across the healthy adult lifespan. Specifically, the FIDL demonstrated a U‐shaped relationship with age, whereby younger and older adults reported lower levels of flexibility, while peak flexibility was reported in middle age (~45–50 years).
- The FIDL provides a new validated tool to assess individual differences in multidimensional expressions of flexibility in daily life. We propose that the FIDL provides a much‐needed clinical research tool that can be used transdiagnostically to detect subtle shifts in flexibility in health and disease.

## INTRODUCTION

The capacity to adjust our thinking and behaviour in response to changes in the environment is an adaptive feature of human cognition that bestows immense flexibility in our daily lives (Armbruster et al., 2012; Dajani & Uddin, 2015; Ionescu, 2012; Scott, 1962). This propensity for cognitive and behavioural flexibility varies considerably between individuals and is suggested to undergo dynamic shifts across the lifespan (Anderson, 2002; Cepeda et al., 2001; Uddin, 2021) manifesting in a diversity of thoughts and behaviours. For example, prior work has documented a U‐shaped relationship between age and one facet of flexibility (task switching), with greater switching capacity evident in middle age relative to younger and older participants (Cepeda et al., 2001; Kupis et al., 2021). Contemporary theories increasingly view cognitive and behavioural flexibility as trait level constructs that can be situated along a continuum (Morris & Mansell, 2018), the extremes of which represent maladaptive instances of entrenched rigidity on the one hand to unconstrained hyper‐flexible thoughts and behaviours on the other (Armbruster et al., 2012; Uddin, 2021). Cognitive and behavioural inflexibility is a transdiagnostic feature of many neuropsychiatric disorders (Uddin, 2021), such as autism spectrum disorder (ASD) and attention‐deficit/hyperactivity disorder (ADHD; Dajani et al., 2016), traumatic brain injury (Pang et al., 2016; Whiting et al., 2017) and neurodegenerative disease (Bozeat et al., 2000; Horne et al., 2023; Robbins & Cools, 2014; Townley et al., 2020). Therefore, efforts to improve the psychometric assessment of these constructs at a trait level represent a nascent topic in the field.

To date, formal assessment of cognitive flexibility has largely centred on the use of standard neuropsychological paradigms that probe the aspects of task switching (Braem & Egner, 2018; Rubinstein et al., 2001), set shifting (Hedden & Gabrieli, 2010; Wager et al., 2004), or reversal learning (Kehagia et al., 2010). The defining feature of such tasks is the requirement to switch between two mental sets, either in response to feedback or in response to explicit instruction. While informative in a clinical context, by focusing on isolated aspects of flexibility, these tasks fail to capture the full gamut of flexible thoughts and behaviours that are displayed across different contexts. This limits the ecological validity of existing tasks as performance on such measures does not necessarily correspond to flexible and inflexible expressions of thinking and behaviour as expressed in daily life (Dang et al., 2020; Geurts et al., 2009). Given that cognitive flexibility tasks also rely heavily on other executive functions such as inhibition and working memory (Buchsbaum et al., 2005; Dajani & Uddin, 2015; Hartman et al., 2001; Sánchez‐Cubillo et al., 2009), the specificity of such tasks is further contentious. This is particularly true for task‐ and set‐switching paradigms which require the maintenance of two or more rules in working memory and successful inhibition of the inappropriate response set (Dajani & Uddin, 2015). As most previous investigations into age‐related changes in flexibility have relied on these paradigms (Anderson, 2002; Cepeda et al., 2001; Kupis et al., 2021), our understanding of cognitive and behavioural flexibility across the lifespan remains limited.

A growing body of evidence suggests that flexibility is multidimensional in nature (Barbey et al., 2013; Steinmetz et al., 2011; Strang et al., 2017) and potentially varies along several underlying factors, such as the preference for routine, restricted interests and insistence on sameness (Schultz & Searleman, 2002; Strang et al., 2017; Uljarević et al., 2023). As a result, several self‐ and informant‐report questionnaires have been developed to capture discrete dimensions of flexibility in daily life. For instance, repetitive and inflexible behaviours in children and adolescents with autism spectrum disorder (ASD) are commonly assessed via carer‐report using the Flexibility Scale (Strang et al., 2017) and the Dimensional Assessment of Repetitive Behaviour scale (DARB; Uljarević et al., 2023), while the Repetitive Behaviours Scale – Revised (Bodfish et al., 2000) measures repetitive behaviours in ASD from early childhood to middle age. However, each of these measures was designed specifically for use in ASD, rendering them unsuitable for broader transdiagnostic use.

Other measures, such as the Cognitive Flexibility Inventory (CFI; Dennis & Vander Wal, 2010) and the Cognitive Control and Flexibility Questionnaire (Gabrys et al., 2018), assess the ability to challenge and adapt maladaptive thoughts in adulthood, specifically in the context of stress. In contrast, the “Shift” scales on the Behaviour Rating Inventory of Executive Functioning (BRIEF, Gioia et al., 2015) and BRIEF Adult version (BRIEF‐A; Roth & Gioia, 2005) have been validated across a broad range of neuropsychiatric conditions such as neurodevelopmental disorders, acquired brain injury, and neurodegeneration. However, these questionnaires are short unidimensional scales which are insufficient to parse the inherent heterogeneity of flexibility across the adult lifespan. As such, there is a crucial need for validated tools that can be applied transdiagnostically to capture individual differences in multidimensional flexibility and to identify alterations from normative function in heterogeneous mental disorders.

To this end, we developed a novel self‐report questionnaire, entitled the Flexibility in Daily Life scale (FIDL). An extensive list of items was reviewed from existing tools within the neuropsychiatric literature. In consultation with a team of experts in neuropsychiatry, psychology, and neurology, 37 items were generated, which aimed to capture common themes across existing scales, in addition to frequent clinical observations. The current validation study investigates the psychometric properties of the FIDL in a large sample of healthy adults from 19 to 78 years old, including factor structure, reliability, and convergent validity, resulting in a final 21‐item scale. In addition, the relationship between the FIDL and age is explored to determine whether the scale is sensitive to natural variations in flexibility across the adult lifespan.

## METHODS

### Participants

A total of 354 participants were recruited through Amazon Mechanical Turk using CloudResearch (formerly TurkPrime; Hauser et al., 2022; Litman et al., 2017; Litman & Robinson, 2020). Recruitment was stratified between four age groups (18–25, 26–40, 41–59, and 60+) with ~90 participants per group to capture the entire adult lifespan. Data quality was optimized using CloudResearch's Approved Participants (Hauser et al., 2022). The final sample included 295 participants following exclusions (Sex: 164 F, 129 M, 2 undisclosed; Mean age = 42.75, *SD* = 17.34, Range = 19–78).

Exclusion criteria included scoring <22 on an online version of the Mini‐ACE (Hsieh et al., 2013), a general screen for cognitive impairment or sub‐optimal attention, or where responses indicated automated completion by a bot (e.g., electronic signatures inserted into clock drawing space; *n* = 47). An additional 12 participants were excluded due to self‐reported history of an acquired brain injury or a neurological condition. All participants were residents of the United States of America.

Participants were monetarily reimbursed for their time ($3.00 USD). The study was approved by the local Ethics Committee (Approval number: 2021/873). All participants provided informed consent in accordance with the Declaration of Helsinki.

### Procedures

#### Item development

An initial pool of 216 items was obtained following a review of existing questionnaires measuring cognitive and/or behavioural flexibility in neuropsychiatric populations (e.g., obsessive compulsive disorder, depression, neurodevelopmental disorders, and neurodegenerative disease). Questionnaires were included in this review if the overall questionnaire or one or more sub‐scales specifically targeted cognitive or behavioural flexibility, or conversely, rigid, restricted, and/or repetitive patterns of behaviour. By leveraging tools from neuropsychiatry, we could ensure that we captured maladaptive expressions of inflexibility, which are characteristic features of neuropsychiatric disorders (Uddin, 2021). The inclusion of neuropsychiatric populations was also necessarily given the current lack of measures assessing daily manifestations of cognitive and behavioural flexibility in healthy populations. Importantly, the Cognitive Flexibility Index (CFI; Dennis & Vander Wal, 2010) was not included in this initial search as this measure was selected for subsequent convergent validity analyses and its inclusion would result in inflated validity estimates. We also elected not to include the Cognitive Control and Flexibility Questionnaire (Gabrys et al., 2018) as it measures the ability to control intrusive thoughts and emotions and to flexibly cope with stressful life events rather than providing a trait level assessment of flexibility. Where scales captured multiple constructs such as social cognition and sensory preferences, only the items measuring flexibility were extracted. A full list of the reviewed questionnaires is provided in Table 1.

**TABLE 1:** Questionnaires reviewed in FIDL item development.

Scale	Number of items extracted	References
BRIEF‐A: “Shift” sub‐scale	6	Roth and Gioia (2005)
CBI‐R: “Stereotypic and Motor Behaviours” Scale	5	Wear et al. (2008)
Repetitive Behaviours Scale‐Revised	22	Bodfish et al. (2000)
Cognitive Flexibility Scale	6	Martin and Rubin (1995)
Flexibility Scale	27	Strang et al. (2017)
Autism Quotient	21	Baron‐Cohen et al. (2001)
AdAS Spectrum: “Inflexibility and adherence to routine” scale	63	Dell'Osso et al. (2017)
DARB	67	Uljarević et al. (2023)

*Note*: Questionnaires reviewed in the item development process are listed alongside the number of items extracted for the current study.

Abbreviations: AdAS, Adult Autism Subthreshold Spectrum; BRIEF‐A, Behaviour Rating Inventory of Executive Function, Adult version; CBI‐R, Cambridge Behavioural Inventory Revised; DARB, Dimensional Assessment of Repetitive Behaviour.

#### Multidisciplinary consensus

First, the initial 216 items were reviewed by the research team, following which 30 items capturing common themes within the flexibility literature were identified (e.g., insistence on sameness, preference for routine, intense interests, rigid ideas and opinions). Questions were re‐written in a combination of positive and negative syntax and phrased in simple terms with appropriate examples to ensure suitability for a wide range of clinical populations and cultures.

The shortlist of 30 items was then presented for discussion at an international consortium on behavioural changes in neuropsychiatric and neurodegenerative populations. Consortium members include neurologists, psychiatrists, and psychologists, from 14 countries. Following multidisciplinary discussion, a further 7 items were included to map onto common clinical observations of cognitive and behavioural inflexibility in neuropsychiatric and neurodegenerative populations. This resulted in a final set of 37 unique items, which was taken forward for validation (see Supporting Information for full item list).

#### Validation procedure

The study was conducted online via Qualtrics software (Provo, Utah, USA) and took approximately 20 minutes to complete. After providing informed consent, participants completed the FIDL and the Cognitive Flexibility Inventory (CFI; Dennis & Vander Wal, 2010) in a randomized order. All questions were self‐paced. Participants then completed the online Mini‐ACE (Hsieh et al., 2013) for screening purposes (see above exclusion criteria). All data were collected between June and August 2023.

When completing the FIDL, participants were asked to consider each statement in relation to the previous two weeks and to indicate the frequency with which each item had occurred on a 5‐point Likert scale (“Never”, “Rarely”, “Sometimes”, “Often”, “All the time”). Each item was scored from 1 to 5, with higher scores indicating less flexibility. Positively worded items were reverse scored.

To establish the convergent validity of the FIDL, participants also completed the CFI as one of the most widely used and representative measures of cognitive flexibility in healthy adults. The CFI consists of 20 items designed to capture individual differences in cognitive flexibility in the context of stressful life events (e.g., “I consider multiple options before making a decision.”). Although the FIDL is designed to capture flexibility in a broader range of settings, the CFI was considered appropriate for use as a convergent validity measure because it measures a highly similar construct, which we would theoretically expect to be related to the FIDL. The CFI also comprises two subscales, measuring the ability to perceive multiple alternative explanations for life occurrences and to generate multiple alternative solutions to difficult situations (“Alternatives”), as well as the tendency to perceive difficult situations as somewhat controllable (“Control”). Participants responded to each item on a 7‐point Likert scale (“Strongly disagree” to “Strongly agree”). Items were scored from 1 to 7, with higher scores indicating a more flexible style of thinking.

### Statistical analysis

Data analyses were performed in R programming software (R Core Team, 2013) using the *psych* (Revelle & Revelle, 2015) and *Hmisc* (Harrell & Dupont, 2020) software packages. As full datasets are required for factor analysis, multiple imputation was used to generate a complete dataset, employing the *mice* R package (Van Buuren & Groothuis‐Oudshoorn, 2011). The original dataset had just .1% missing data.

Bartlett's test of Sphericity (Bartlett, 1950) and the Kaiser–Meyer–Olkin test (KMO, Kaiser, 1974) were used to evaluate the factorability of the data. Horn's parallel analysis of principal factors (Horn, 1965) was used to determine the optimal number of factors for extraction. This approach is widely recognized as the gold standard in factor analysis research (Frazier & Youngstrom, 2007; Hoelzle & Meyer, 2012; Velicer et al., 2000; Velicer & Fava, 1998; Watkins, 2018) and is preferable to alternative approaches such as Kaiser's criterion, which is more closely related to the number of variables analysed, rather than the underlying structure of the data (Fabrigar & Wegener, 2011; Hoelzle & Meyer, 2012; Izquierdo et al., 2014; Norris & Lecavalier, 2010).

An iterated principal axis exploratory factor analysis (EFA) was run using the promax (oblique) rotation method with an a priori factor loading cut‐off of ≥.40 and a cross‐loading cut‐off of ≥.30 (Howard, 2016). An oblique rotation method was selected as we expected the factors to be correlated (Fabrigar & Wegener, 2011; Howard, 2016; Watkins, 2018). This step was repeated iteratively with the remaining items to determine the final factor structure.

The internal consistency of the resulting questionnaire was evaluated by calculating average inter‐item and item‐scale correlations, and Cronbach's alpha, for each factor and the overall FIDL score. In addition, split‐half reliability was assessed by calculating Pearson's correlation between odd and evenly numbered items. To assess the convergent validity of the FIDL, correlations were computed between each FIDL factor and total FIDL score, with the CFI Total, Alternatives and Control scales. Pearson correlations or Spearman's Rho were used, depending on the multivariate normality. *p*‐values were adjusted for multiple comparisons using the Benjamini‐Hochberg procedure (Benjamini & Hochberg, 1995), with a critical alpha level of .05.

Quadratic regression was used to determine the relationship between the FIDL total score and each subscale score with age, as visual inspection indicated that the data followed a U‐shaped curve. The quadratic regression model was compared to the linear regression model to ensure that the quadratic term added significant variance to the model.

## RESULTS

### Exploratory factor analysis

Bartlett's test of Sphericity (*χ* ^2^[666] = 4526.88, *p* < .001) indicated that the correlation matrix was not random, and the KMO statistic (.89) exceeded the recommended minimum (≥.70; Hoelzle & Meyer, 2012; Lloret et al., 2017). Therefore, the 37‐item questionnaire was considered appropriate for factor analysis and was submitted for EFA.

The initial EFA yielded a six‐factor solution, as indicated by Horn's parallel analysis, which accounted for 45% of the total variance. Items with factor loadings <.4, and/or cross‐loadings ≥.30 were removed, and the EFA was repeated with the remaining items. This process was repeated twice to derive the final five‐factor structure which accounted for 49% of the total variance. The final solution consisted of 21 items with all loadings ≥.4 (see Supporting Information for revised questionnaire). Eigenvalues and the proportion of variance accounted for by each factor are presented in Table 2.

**TABLE 2:** Exploratory factor analysis solution.

Factor number	Label	Eigenvalue	Proportion variance	Cumulative variance	Number of items
1	Repetition	5.78	.14	.14	6
2	Switching	1.97	.11	.25	5
3	Predictability/Control	1.02	.10	.36	4
4	Routine	.92	.07	.43	3
5	Thoughts/Beliefs	.67	.07	.49	3

*Note*: Five‐factor solution and labels from principal axis exploratory factor analysis with promax (oblique) rotation.

Each factor was assigned a label based on the overarching theme across its respective items. Factor 1 was labelled “*Repetition*”, reflecting repetitive and/or stereotypical behaviours and interests (e.g., “I need to repeat some actions/tasks until they feel ‘just right’”). Factor 2, named “*Switching*”, captured the ability to shift or adapt one's behaviour or mental set in response to feedback or changes in their environment (e.g., “I can easily change my approach if shown a better way”). Factor 3 was labelled “*Predictability/Control*”, with items reflecting a need for structure and order in situations and/or others' behaviour (e.g., “I am very unsettled by last‐minute changes to my plans.”). Factor 4, labelled “*Routine*”, described a preference for specific routines (e.g., “I like to follow specific routines when I leave the house…”). Finally, Factor 5, named “*Thoughts/Beliefs*”, captured rigid or black‐and‐white thinking styles and beliefs (e.g., “I find it hard to let go of my ideas or beliefs, even when presented with new information.”).

### Internal consistency reliability

The total FIDL score showed good internal consistency (Cronbach's alpha, *α* = .88) and moderate‐to‐good internal consistency for each of the subscales (*Repetition*: *α* = .84; *Switching*: *α* = .80; *Predictability/Control*: *α* = .78; *Routine*: *α* = .70; *Thoughts/Beliefs*: *α* = .69). Split‐half reliability of the overall measure was strong (*r* = .86, *p* < .001). Mean inter‐item correlations were moderate for all five subscales (*Repetition*: Mean *r* = .46; *Switching*: Mean *r* = .44; Predictability: Mean *r* = .46; *Routine*: Mean *r* = .44; *Thoughts/Beliefs*: Mean *r* = .43) falling within recommended guidelines (.15 to .50; Clark & Watson, 2019). The average inter‐item correlation for the overall measure was .26, with moderate item‐scale correlations across the subscales (*Repetition*: *r* = .61; *Switching*: *r* = .58; *Predictability/Control*: *r* = .59; *Routine*: *r* = .52; *Thoughts/Beliefs*: *r* = .51). The average item‐scale correlation for the overall measure was also moderate (*r* = .47). Overall, these metrics demonstrate good internal consistency reliability for each individual factor and the total FIDL scale.

### Convergent validity

Correlations between the total and individual factor scores of the FIDL and the CFI are presented in Table 3. The FIDL displayed good overall convergent validity with the CFI, as reflected by significant negative correlations between the Total FIDL and CFI Total, Control and Alternatives scales (all *p* values < .05), such that greater scores on the FIDL (denoting less flexibility), were associated with lower scores on the CFI (reflecting lower cognitive flexibility). Similarly, all FIDL Factor scores were significantly negatively correlated with the CFI total score and the CFI Control scale. However, only the *Switching* and *Thoughts/Beliefs* factors were significantly negatively correlated with the CFI Alternatives scale. This suggests that the FIDL *Repetition*, *Predictability/Control* and *Routine* scales capture unique aspects of flexibility to the CFI Alternatives scale, possibly due to their emphasis on behavioural rather than cognitive features. In summary, the FIDL displays good convergent validity with discrete aspects of the CFI (i.e. the Total CFI and Control subscale). Importantly, not all factors on the FIDL were associated with the CFI factors, suggesting the FIDL captures a broader range of flexible thoughts and behaviours. This is unsurprising given that the CFI is intended to measure specific aspects of flexibility within specific contexts and displays a two‐factor structure, compared to the comprehensive five‐factor structure of the FIDL.

**TABLE 3:** Correlations between FIDL and CFI scales.

Factor	CFI total	CFI alternatives	CFI control
1. Repetition	−.28**	−.04	−.39**
2. Switching	−.40**	−.30**	−.32**
3. Predictability	−.28**	−.02	−.40**^a^
4. Routine	−.18**	−.05^a^	−.21**^a^
5. Cognitive	−.28**	−.20**	.23**
FIDL total	−.41**	−.15*	−.47**

*Note*: *p < .05; **p < .01; All correlations are Spearman's Rho unless otherwise specified (^a^Pearson's *r*). *p* values are corrected for False Discovery Rate at *q* = .05 using the Benjamini–Hochberg procedure.

Abbreviations: CFI, Cognitive Flexibility Inventory; FIDL, Flexibility in Daily Life scale.

### Association between FIDL total score and age

**FIGURE 1:**
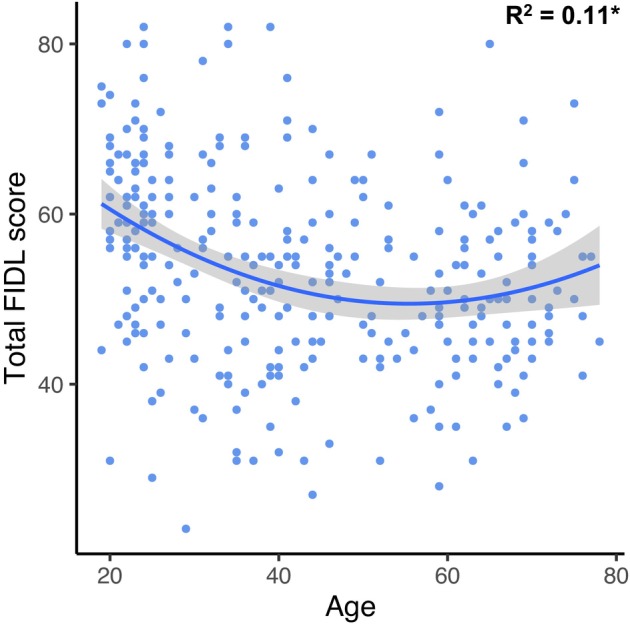
Relationship between total flexibility scores on the FIDL and age. Figure depicts quadratic regression between FIDL total flexibility scores and age, where higher FIDL scores denote lower self‐reported levels of flexibility. Shaded area depicts 95% confidence interval; *p < .001; formula: FIDL = .009(Age)^2^ + −.981(Age) − 76.651.

Finally, we were interested to explore how expressions of flexibility might naturally shift over the course of the adult lifespan. A significant quadratic relationship was found between age and total FIDL (*t* = 3.47, *p* < .001), such that flexibility followed a U‐shaped trajectory across the adult lifespan (see Figure 1). Specifically, self‐reported flexibility was greatest in middle aged adults, with lower overall self‐reported levels of flexibility at the younger and older tails of the lifespan. The model accounted for 11% of the variance, which was significantly greater than the linear regression model without the quadratic term (*F*[1, 292] = 12.05, *p* < .001).

### Quadratic associations between FIDL dimensions and age

**FIGURE 2:**
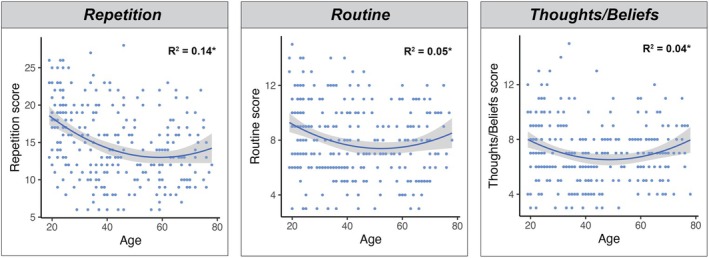
Quadratic associations between age and distinct dimensions of flexibility on the FIDL. Figure depicts quadratic regressions between FIDL Repetition, Routine and Thoughts/Beliefs subscale scores and age; Shaded areas depict 95% confidence intervals; *p < .001; Regression formulas: Repetition = .004 (Age)^2^ + −.410(Age) + 25.104; Routine = .002(Age)^2^ + −.178(Age) + 12.057; Thoughts/Beliefs = .002(Age)^2^ + −.163(Age) + 10.492.

To determine whether distinct dimensions of flexibility underpin the above U‐shaped relationship between age and Total FIDL, further regression analyses were run between age and each FIDL subscale. Quadratic relationships were found exclusively for the *Repetition* (*t* = 3.186, *p* = .002, *r* ^2^ = .137), *Routine* (*t* = 2.837, *p* = .005, *r* ^2^ = .049) and *Thoughts/Beliefs* (*t* = 3.249, *p* = .001, *r* ^2^ = .041) factors (see Figure 2) whereby self‐reported flexibility was greatest in middle age and lower at the younger and older tails of the adult lifespan.

Interestingly, we observed subtle variations in the trajectory of these changes across individual FIDL subscales. Specifically, the *Thoughts/Beliefs* and *Routine* factors were lowest (denoting greater flexibility) at approximately 45 years of age. This suggests that individuals are most open to alternate ideas and viewpoints and least insistent on routines during middle age. In contrast, lower scores on the *Repetition* subscale occurred slightly later (~50 years), reflecting the age at which individuals are least likely to seek out repetitive and stereotypical behaviours. These factors underwent a progressive shift towards lower flexibility from approximately 50 years of age for the *Thoughts/Beliefs* and *Routine* factors, and at approximately 60 years for the *Repetition* scale.

### Linear associations between FIDL dimensions and age

**FIGURE 3:**
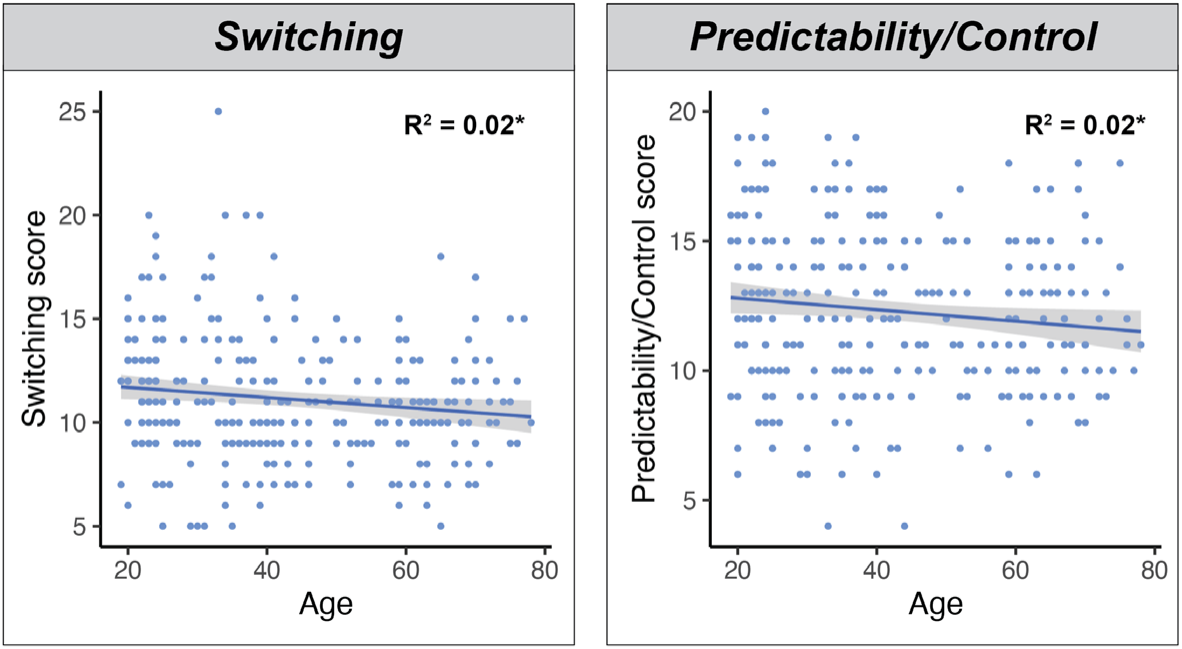
FIDL subscales demonstrating linear associations with age. Figure depicts linear regressions between FIDL Switching and Predictability/Control subscale scores and age. Shaded areas depict 95% confidence intervals; *p < .001; Regression formula: Switching = −.024(Age) + 12.183; Predictability/Control = −.022(Age) + 13.241.

In contrast, the quadratic term was non‐significant for the *Switching* (*t* = 1.446, *p* > .05) and *Predictability/Control* (*t* = 1.337, *p* > .05) factors (see Figure 3), which were better characterized by a linear relationship. Specifically, both factors displayed a significant negative association with age (*Switching*: *β* = −.02, *p* = .017, *r* ^2^ = .019; *Predictability/Control*: *β* = −.02, *p* = .035, *r* ^2^ = .015), such that self‐reported flexibility increased across the lifespan. This finding suggests that as individuals get older, they report slight improvements in their ability to adapt their task or mental set, along with a reduced need for predictability and order in their daily life. However, it is important to note that both regressions were characterized by a gradual slope, suggesting only subtle changes in these factors across the lifespan.

## DISCUSSION

Flexibility of thought and behaviour is crucial for adaptive functioning and appears to be compromised across a wide array of neuropsychiatric and neurological disorders (Uddin, 2021). Progress in this field has been hampered by a lack of validated empirical tools that are sensitive to natural variations in multidimensional flexibility and can be used transdiagnostically. Here, we present the FIDL as a robust new measure that effectively captures individual differences in the diversity of flexible thoughts and behaviour that manifest in daily life. Our findings indicate that the FIDL is a valid and reliable measure, with a clearly defined factor structure, and is sensitive to naturally occurring changes in flexibility across the adult lifespan. Importantly, we demonstrate that subcomponents of flexibility, as indexed by the FIDL, follow distinct trajectories across different timepoints of the adult lifespan, providing critical windows from which deviations from normative function might be tracked.

Considering first the psychometric properties of the FIDL, an exploratory factor analysis produced a revised 21‐item scale with a five‐factor solution. The emergent factors captured the following constructs: repetitive and stereotypical behaviours (*Repetition*), the ability to adapt one's task or mental set in response to changes in the environment or feedback (*Switching*), the preference for structured and ordered environments or social settings (*Predictability/Control*), the preference for routine (*Routine*), and rigid thinking styles or beliefs (*Thoughts/Beliefs*). The 21‐item FIDL exhibited good to strong internal consistency, whereas all sub‐scales had moderate to strong internal consistency. Furthermore, associations between the FIDL total and individual subscales and an established measure of cognitive flexibility, the CFI (Dennis & Vander Wal, 2010), further provided evidence for the convergent validity of the new scale. Although the correlations observed were relatively modest, all associations ran in the predicted direction, suggesting that discrete dimensions of flexibility as indexed by the FIDL correspond to the Control and, to a lesser extent, Alternatives subscales of the CFI. This is unsurprising given that the FIDL incorporates core aspects of cognitive flexibility which form the cornerstone of the CFI, such as the ability to consider multiple viewpoints and think flexibly about situations. Importantly, however, the FIDL enriches the assessment of flexibility by incorporating additional behavioural features, such as repetitive and stereotyped behaviours, whilst sampling a broader range of cognitive aspects such as the presence of rigid and unchanging thoughts and beliefs, providing a more comprehensive assessment of multidimensional flexibility.

A notable finding of our study was that overall flexibility followed a U‐shaped curve across the adult lifespan, with lower flexibility at either tail of the distribution. Self‐reported flexibility was observed to increase from early to middle adulthood (~40 years), following which it progressively approached a more inflexible style of interacting with the world in older adulthood (~60 years). This trajectory is broadly consistent with previous work demonstrating a u‐shaped relationship between age and switching task performance (Cepeda et al., 2001; Kupis et al., 2021; Schultz & Searleman, 2002; Uddin, 2021), providing further support for the construct validity of the FIDL. Considerable variability has been reported in the literature in terms of the relationship between flexibility and age. For example, some studies have found a linear decline in task switching abilities with age (Kray & Lindenberger, 2000; Wecker et al., 2005), or simply that older adults are less adept at overcoming existing cognitive biases when compared to younger adults (Wilson et al., 2018). In contrast, meta‐analytic research has demonstrated no evidence for an effect of age on task‐switching costs after accounting for general slowing (Chen & Hsieh, 2023). As such, it has been suggested that the manner by which flexibility is assessed has considerable bearing on the results elicited (Chen & Hsieh, 2023). These mixed findings likely also reflect the multidimensional nature of flexibility, whereby discrete components of this construct may be differentially affected by the aging process. Our finding of distinct age‐related trajectories on individual subscales of the FIDL suggests this, indeed, may be the case. A gradual reduction in flexibility in older age resonates well with the shifting mental mode framework, which proposes a bias towards exploitative rather than exploratory decision‐making as the foundation for all forms of thought and behaviour in healthy aging (Spreng & Turner, 2021). Accordingly, the predisposition towards exploitation in older adults manifests in a bias towards less flexible forms of thinking and behaviour.

Notably, our findings suggest that this age‐related transition towards a more exploitative mental mode does not occur uniformly across all dimensions of flexibility. Using the FIDL we could estimate the onset of distinct shifts in flexibility across the adult lifespan. Critically, of the three dimensions to display a U‐shaped relationship with age, we observed distinct shifts at different temporal timescales for each of the *Repetition*, *Routine*, and *Thoughts/Beliefs* factors. The earliest shift in mental mode was found to occur at ~50 years (*Thoughts/Beliefs*, *Routine*), followed by a second shift at ~60 years in which repetitive behaviours became more prominent (*Repetition*). Collectively, these subscales provide important information regarding expected natural shifts towards exploitative modes of thinking and behaviour, from which departures from normative function can be tracked.

From a clinical perspective, the FIDL can be used to reconcile several conflicting findings in the literature. First, we demonstrate the need to deconstruct the overall construct of flexibility into distinct subcomponents, each of which likely follows separable trajectories across the adult lifespan. The inherent multidimensionality of flexibility likely can account for some of the previous challenges in conceptualization and assessment of this construct (Dajani & Uddin, 2015; Uddin, 2021), particularly the poor agreement between neuropsychological tasks and existing questionnaires (Dang et al., 2020; Geurts et al., 2009; Uddin, 2021). For instance, the FIDL *Switching* factor describes the ability to switch between mental sets in response to a change in the environment or feedback, and aligns well with the type of flexibility assessed in neuropsychological task‐ and set‐shifting paradigms. However, our results indicate that this dimension is separable from other behavioural manifestations of inflexibility in daily life such as the insistence on routine, or the preference for predictability and familiarity. This multi‐dimensional framework could also explain paradoxical findings of inflexibility alongside distractibility in clinical populations, as is often the case in comorbid ASD and attention‐deficit/hyperactivity disorder (ADHD; Uddin, 2021).

One limitation of the current study is that we did not validate the measure in a second, independent sample. Future work using confirmatory factor analysis (CFA) in a new independent sample will be essential to corroborate the factor structure reported here. Additionally, as the current validation study was limited to a healthy adult population, a crucial next step will be to validate the FIDL in clinical populations in which inflexibility is reported. Such populations include, but are not limited to, ASD and ADHD (Dajani et al., 2016), traumatic brain injury (Pang et al., 2016; Whiting et al., 2017), neurodegenerative disease (e.g. frontotemporal dementia [FTD], Parkinson's disease, Alzheimer's disease; Bozeat et al., 2000; Horne et al., 2023; Robbins & Cools, 2014; Townley et al., 2020), and obsessive‐compulsive disorder (OCD; Akkermans et al., 2019; Gruner & Pittenger, 2017). Such validation studies will determine whether the dimensional architecture of flexibility differs across disease aetiologies, and will facilitate the establishing of clinical cut‐off points to determine maladaptive expressions of inflexibility. Finally, although the current version of the FIDL is designed exclusively for use in adulthood, we note the potential to modify the measure for use in childhood or adolescence. This would enable us to adopt a whole‐of‐life approach to trajectories of flexibility and to chart its course longitudinally.

In summary, the FIDL is a novel and validated measure of multidimensional cognitive and behavioural flexibility suitable for use across the adult lifespan. The scale upholds good psychometric properties, is sensitive to natural fluctuations in flexibility across the adult lifespan, and suitable for use transdiagnostically. Integration of the FIDL into neuropsychological test batteries at large will enable the creation of reliable thresholds from which departures from normative function can be determined. This represents a crucial next step to map the underlying cognitive and neural determinants of disease‐specific profiles of inflexibility, to understand their functional implications, and to ultimately arrive at a comprehensive taxonomy of flexibility in health and disease.

## AUTHOR CONTRIBUTIONS

**Kristina Horne:** Conceptualization; data curation; formal analysis; writing – original draft; methodology. **Tao Chen:** Conceptualization; writing – review and editing; methodology. **Muireann Irish:** Conceptualization; writing – original draft; methodology; supervision; writing – review and editing; funding acquisition; resources.

## CONFLICT OF INTEREST STATEMENT

This authors report no conflicts of interest.

## Supporting information


Appendix S1.

## Data Availability

The data that support the findings of this study will be made openly available in a public repository prior to publication.

## References

[bjc12505-bib-0001] Akkermans, S. E. A. , Rheinheimer, N. , Bruchhage, M. M. K. , Durston, S. , Brandeis, D. , Banaschewski, T. , Boecker‐Schlier, R. , Wolf, I. , Williams, S. C. R. , Buitelaar, J. K. , van Rooij, D. , & Oldehinkel, M. (2019). Frontostriatal functional connectivity correlates with repetitive behaviour across autism spectrum disorder and obsessive‐compulsive disorder. Psychological Medicine, 49(13), 2247–2255. 10.1017/s0033291718003136 30362446

[bjc12505-bib-0002] Anderson, P. (2002). Assessment and development of executive function (EF) during childhood. Child Neuropsychology, 8(2), 71–82. 10.1076/chin.8.2.71.8724 12638061

[bjc12505-bib-0003] Armbruster, D. J. , Ueltzhöffer, K. , Basten, U. , & Fiebach, C. J. (2012). Prefrontal cortical mechanisms underlying individual differences in cognitive flexibility and stability. Journal of Cognitive Neuroscience, 24(12), 2385–2399. 10.1162/jocn_a_00286 22905818

[bjc12505-bib-0004] Barbey, A. K. , Colom, R. , & Grafman, J. (2013). Architecture of cognitive flexibility revealed by lesion mapping. NeuroImage, 82, 547–554. 10.1016/j.neuroimage.2013.05.087 23721727 PMC3790579

[bjc12505-bib-0005] Baron‐Cohen, S. , Wheelwright, S. , Skinner, R. , Martin, J. , & Clubley, E. (2001). The autism‐spectrum quotient (AQ): Evidence from Asperger syndrome/high‐functioning autism, males and females, scientists and mathematicians. Journal of Autism and Developmental Disorders, 31(1), 5–17. 10.1023/a:1005653411471 11439754

[bjc12505-bib-0006] Bartlett, M. S. (1950). Tests of significance in factor analysis. British Journal of Psychology, 3, 77–85. 10.1111/j.2044-8317.1950.tb00285.x

[bjc12505-bib-0007] Benjamini, Y. , & Hochberg, Y. (1995). Controlling the false discovery rate: A practical and powerful approach to multiple testing. Journal of the Royal Statistical Society: Series B: Methodological, 57(1), 289–300. 10.1111/j.2517-6161.1995.tb02031.x

[bjc12505-bib-0008] Bodfish, J. W. , Symons, F. J. , Parker, D. E. , & Lewis, M. H. (2000). Repetitive behavior scale–revised (RBS‐R) [Database record]. APA PsycTests. 10.1037/t17338-000

[bjc12505-bib-0009] Bozeat, S. , Gregory, C. A. , Ralph, M. A. , & Hodges, J. R. (2000). Which neuropsychiatric and behavioural features distinguish frontal and temporal variants of frontotemporal dementia from Alzheimer's disease? Journal of Neurology, Neurosurgery, and Psychiatry, 69(2), 178–186. 10.1136/jnnp.69.2.178 10896690 PMC1737062

[bjc12505-bib-0010] Braem, S. , & Egner, T. (2018). Getting a grip on cognitive flexibility. Current Directions in Psychological Science, 27(6), 470–476. 10.1177/0963721418787475 30555214 PMC6291219

[bjc12505-bib-0011] Buchsbaum, B. R. , Greer, S. , Chang, W. L. , & Berman, K. F. (2005). Meta‐analysis of neuroimaging studies of the Wisconsin card‐sorting task and component processes. Human Brain Mapping, 25(1), 35–45. 10.1002/hbm.20128 15846821 PMC6871753

[bjc12505-bib-0012] Cepeda, N. J. , Kramer, A. F. , & Gonzalez de Sather, J. C. (2001). Changes in executive control across the life span: Examination of task‐switching performance. Developmental Psychology, 37(5), 715–730. 10.1037/0012-1649.37.5.715 11552766

[bjc12505-bib-0013] Chen, E.‐H. , & Hsieh, S. (2023). The effect of age on task switching: Updated and extended meta‐analyses. Psychological Research, 87(7), 2011–2030. 10.1007/s00426-023-01794-z 36729159

[bjc12505-bib-0014] Clark, L. A. , & Watson, D. (2019). Constructing validity: New developments in creating objective measuring instruments. Psychological Assessment, 31(12), 1412–1427. 10.1037/pas0000626 30896212 PMC6754793

[bjc12505-bib-0015] Dajani, D. R. , Llabre, M. M. , Nebel, M. B. , Mostofsky, S. H. , & Uddin, L. Q. (2016). Heterogeneity of executive functions among comorbid neurodevelopmental disorders. Scientific Reports, 6, 36566. 10.1038/srep36566 27827406 PMC5101520

[bjc12505-bib-0016] Dajani, D. R. , & Uddin, L. Q. (2015). Demystifying cognitive flexibility: Implications for clinical and developmental neuroscience. Trends in Neurosciences, 38(9), 571–578. 10.1016/j.tins.2015.07.003 26343956 PMC5414037

[bjc12505-bib-0017] Dang, J. , King, K. M. , & Inzlicht, M. (2020). Why are self‐report and behavioral measures weakly correlated? Trends in Cognitive Sciences, 24(4), 267–269. 10.1016/j.tics.2020.01.007 32160564 PMC7977810

[bjc12505-bib-0018] Dell'Osso, L. , Gesi, C. , Massimetti, E. , Cremone, I. M. , Barbuti, M. , Maccariello, G. , Moroni, I. , Barlati, S. , Castellini, G. , Luciano, M. , Bossini, L. , Rocchetti, M. , Signorelli, M. , Aguglia, E. , Fagiolini, A. , Politi, P. , Ricca, V. , Vita, A. , Carmassi, C. , & Maj, M. (2017). Adult Autism Subthreshold Spectrum (AdAS Spectrum): Validation of a questionnaire investigating subthreshold autism spectrum. Comprehensive Psychiatry, 73, 61–83. 10.1016/j.comppsych.2016.11.001 27918948

[bjc12505-bib-0019] Dennis, J. P. , & Vander Wal, J. S. (2010). The cognitive flexibility inventory: Instrument development and estimates of reliability and validity. Cognitive Therapy and Research, 34, 241–253. 10.1007/s10608-009-9276-4

[bjc12505-bib-0020] Fabrigar, L. R. , & Wegener, D. T. (2011). Exploratory factor analysis (1st ed.). Oxford University Press. 10.1093/acprof:osobl/9780199734177.001.0001

[bjc12505-bib-0021] Frazier, T. W. , & Youngstrom, E. A. (2007). Historical increase in the number of factors measured by commercial tests of cognitive ability: Are we overfactoring? Intelligence, 35(2), 169–182. 10.1016/j.intell.2006.07.002

[bjc12505-bib-0022] Gabrys, R. L. , Tabri, N. , Anisman, H. , & Matheson, K. (2018). Cognitive control and flexibility in the context of stress and depressive symptoms: The cognitive control and flexibility questionnaire. Frontiers in Psychology, 9, 2219. 10.3389/fpsyg.2018.02219 30510530 PMC6252356

[bjc12505-bib-0023] Geurts, H. M. , Corbett, B. , & Solomon, M. (2009). The paradox of cognitive flexibility in autism. Trends in Cognitive Sciences, 13(2), 74–82. 10.1016/j.tics.2008.11.006 19138551 PMC5538880

[bjc12505-bib-0024] Gioia, G. , Isquith, P. , Guy, S. , & Kenworthy, L. (2015). Behavior rating inventory of executive function–second edition (BRIEF2). Psychological Assessment Resources, *2*.

[bjc12505-bib-0025] Gruner, P. , & Pittenger, C. (2017). Cognitive inflexibility in obsessive‐compulsive disorder. Neuroscience, 345, 243–255. 10.1016/j.neuroscience.2016.07.030 27491478 PMC5288350

[bjc12505-bib-0026] Harrell, F. E. , & Dupont, C. (2020). Hmisc: Harrell miscellaneous. *R package version*, *4*(0).

[bjc12505-bib-0027] Hartman, M. , Bolton, E. , & Fehnel, S. E. (2001). Accounting for age differences on the Wisconsin card sorting test: Decreased working memory, not inflexibility. Psychology and Aging, 16(3), 385–399. 10.1037/0882-7974.16.3.385 11554518

[bjc12505-bib-0028] Hauser, D. J. , Moss, A. J. , Rosenzweig, C. , Jaffe, S. N. , Robinson, J. , & Litman, L. (2022). Evaluating CloudResearch's approved group as a solution for problematic data quality on MTurk. Behavior Research Methods, 55, 3953–3964. 10.3758/s13428-022-01999-x 36326997 PMC10700412

[bjc12505-bib-0029] Hedden, T. , & Gabrieli, J. D. (2010). Shared and selective neural correlates of inhibition, facilitation, and shifting processes during executive control. NeuroImage, 51(1), 421–431. 10.1016/j.neuroimage.2010.01.089 20123030 PMC2852172

[bjc12505-bib-0030] Hoelzle, J. B. , & Meyer, G. J. (2012). Exploratory factor analysis: Basics and beyond. In Handbook of psychology (Vol. 2, 2nd ed.). Wiley. 10.1002/9781118133880.hop202006

[bjc12505-bib-0031] Horn, J. L. (1965). A rationale and test for the number of factors in factor analysis. Psychometrika, 30(2), 179–185. 10.1007/BF02289447 14306381

[bjc12505-bib-0032] Horne, K. , Ahmed, R. M. , Piguet, O. , & Irish, M. (2023). Establishing the link between motivational disturbances and behavioural rigidity in frontotemporal dementia. European Journal of Neurology, 31, e16132. 10.1111/ene.16132 37933881 PMC11235754

[bjc12505-bib-0033] Howard, M. C. (2016). A review of exploratory factor analysis decisions and overview of current practices: What we are doing and how can we improve? International Journal of Human‐Computer Interaction, 32(1), 51–62. 10.1080/10447318.2015.1087664

[bjc12505-bib-0034] Hsieh, S. , Schubert, S. , Hoon, C. , Mioshi, E. , & Hodges, J. R. (2013). Validation of the Addenbrooke's cognitive examination III in frontotemporal dementia and Alzheimer's disease. Dementia and Geriatric Cognitive Disorders, 36(3–4), 242–250. 10.1159/000351671 23949210

[bjc12505-bib-0035] Ionescu, T. (2012). Exploring the nature of cognitive flexibility. New Ideas in Psychology, 30(2), 190–200. 10.1016/j.newideapsych.2011.11.001

[bjc12505-bib-0036] Izquierdo, I. , Olea, J. , & Abad, F. J. (2014). Exploratory factor analysis in validation studies: Uses and recommendations. Psicothema, 26(3), 395–400. 10.7334/psicothema2013.349 25069561

[bjc12505-bib-0037] Kaiser, H. F. (1974). An index of factorial simplicity. Psychometrika, 39(1), 31–36. 10.1007/BF02291575

[bjc12505-bib-0038] Kehagia, A. A. , Murray, G. K. , & Robbins, T. W. (2010). Learning and cognitive flexibility: Frontostriatal function and monoaminergic modulation. Current Opinion in Neurobiology, 20(2), 199–204. 10.1016/j.conb.2010.01.007 20167474

[bjc12505-bib-0039] Kray, J. , & Lindenberger, U. (2000). Adult age differences in task switching. Psychology and Aging, 15(1), 126–147. 10.1037/0882-7974.15.1.126 10755295

[bjc12505-bib-0040] Kupis, L. , Goodman, Z. T. , Kornfeld, S. , Hoang, S. , Romero, C. , Dirks, B. , Dehoney, J. , Chang, C. , Spreng, R. N. , & Nomi, J. S. (2021). Brain dynamics underlying cognitive flexibility across the lifespan. Cerebral Cortex, 31(11), 5263–5274. 34145442 10.1093/cercor/bhab156 PMC8491685

[bjc12505-bib-0041] Litman, L. , & Robinson, J. (2020). Conducting online research on Amazon mechanical Turk and beyond. Sage Publications.

[bjc12505-bib-0042] Litman, L. , Robinson, J. , & Abberbock, T. (2017). TurkPrime.Com: A versatile crowdsourcing data acquisition platform for the behavioral sciences. Behavior Research Methods, 49(2), 433–442. 10.3758/s13428-016-0727-z 27071389 PMC5405057

[bjc12505-bib-0043] Lloret, S. , Ferreres, A. , Hernández, A. , & Tomás, I. (2017). The exploratory factor analysis of items: Guided analysis based on empirical data and software. Anales de Psicología, 33(2), 417–432. 10.6018/analesps.33.2.270211

[bjc12505-bib-0044] Martin, M. M. , & Rubin, R. B. (1995). A new measure of cognitive flexibility. Psychological Reports, 76(2), 623–626. 10.2466/pr0.1995.76.2.623

[bjc12505-bib-0045] Morris, L. , & Mansell, W. (2018). A systematic review of the relationship between rigidity/flexibility and transdiagnostic cognitive and behavioral processes that maintain psychopathology. Journal of Experimental Psychopathology, 9(3), 2043808718779431. 10.1177/2043808718779431

[bjc12505-bib-0046] Norris, M. , & Lecavalier, L. (2010). Evaluating the use of exploratory factor analysis in developmental disability psychological research. Journal of Autism and Developmental Disorders, 40(1), 8–20. 10.1007/s10803-009-0816-2 19609833

[bjc12505-bib-0047] Pang, E. W. , Dunkley, B. T. , Doesburg, S. M. , da Costa, L. , & Taylor, M. J. (2016). Reduced brain connectivity and mental flexibility in mild traumatic brain injury. Annals of Clinical Translational Neurology, 3(2), 124–131. 10.1002/acn3.280 26900581 PMC4748313

[bjc12505-bib-0048] R Core Team, R . (2013). R: A language and environment for statistical computing.

[bjc12505-bib-0049] Revelle, W. , & Revelle, M. W. (2015). Package ‘psych’. *The comprehensive R archive network*, *337*(338).

[bjc12505-bib-0050] Robbins, T. W. , & Cools, R. (2014). Cognitive deficits in Parkinson's disease: A cognitive neuroscience perspective. Movement Disorders, 29(5), 597–607. 10.1002/mds.25853 24757109

[bjc12505-bib-0051] Roth, R. M. , & Gioia, G. A. (2005). Behavior rating inventory of executive function–adult version. Psychological Assessment Resources.

[bjc12505-bib-0052] Rubinstein, J. S. , Meyer, D. E. , & Evans, J. E. (2001). Executive control of cognitive processes in task switching. Journal of Experimental Psychology: Human Perception and Performance, 27(4), 763–797. 10.1037//0096-1523.27.4.763 11518143

[bjc12505-bib-0053] Sánchez‐Cubillo, I. , Periáñez, J. A. , Adrover‐Roig, D. , Rodríguez‐Sánchez, J. M. , Ríos‐Lago, M. , Tirapu, J. , & Barceló, F. (2009). Construct validity of the trail making test: Role of task‐switching, working memory, inhibition/interference control, and visuomotor abilities. Journal of the International Neuropsychological Society, 15(3), 438–450. 10.1017/S1355617709090626 19402930

[bjc12505-bib-0054] Schultz, P. W. , & Searleman, A. (2002). Rigidity of thought and behavior: 100 years of research. Genetic, Social, and General Psychology Monographs, 128(2), 165–207. 12194421

[bjc12505-bib-0055] Scott, W. A. (1962). Cognitive complexity and cognitive flexibility. Sociometry, 25, 405–414. 10.2307/2785779

[bjc12505-bib-0056] Spreng, R. N. , & Turner, G. R. (2021). From exploration to exploitation: A shifting mental mode in late life development. Trends in Cognitive Sciences, 25(12), 1058–1071. 10.1016/j.tics.2021.09.001 34593321 PMC8844884

[bjc12505-bib-0057] Steinmetz, J.‐P. , Loare, E. , & Houssemand, C. (2011). Rigidity of attitudes and behaviors: A study on the validity of the concept. Individual Differences Research, 9(2), 84–106. https://search.ebscohost.com/login.aspx?direct=true&AuthType=ip,shib&db=asx&AN=66939924&site=eds‐live&custid=s3382554

[bjc12505-bib-0058] Strang, J. F. , Anthony, L. G. , Yerys, B. E. , Hardy, K. K. , Wallace, G. L. , Armour, A. C. , Dudley, K. , & Kenworthy, L. (2017). The flexibility scale: Development and preliminary validation of a cognitive flexibility measure in children with autism spectrum disorders. Journal of Autism and Developmental Disorders, 47(8), 2502–2518. 10.1007/s10803-017-3152-y 28527097

[bjc12505-bib-0059] Townley, R. A. , Graff‐Radford, J. , Mantyh, W. G. , Botha, H. , Polsinelli, A. J. , Przybelski, S. A. , Machulda, M. M. , Makhlouf, A. T. , Senjem, M. L. , Murray, M. E. , Reichard, R. R. , Savica, R. , Boeve, B. F. , Drubach, D. A. , Josephs, K. A. , Knopman, D. S. , Lowe, V. J. , Jack, C. R., Jr. , Petersen, R. C. , & Jones, D. T. (2020). Progressive dysexecutive syndrome due to Alzheimer's disease: A description of 55 cases and comparison to other phenotypes. Brain Communications, 2(1), fcaa068. 10.1093/braincomms/fcaa068 PMC7325839 32671341

[bjc12505-bib-0060] Uddin, L. Q. (2021). Cognitive and behavioural flexibility: Neural mechanisms and clinical considerations. Nature Reviews Neuroscience, 22(3), 167–179. 10.1038/s41583-021-00428-w 33536614 PMC7856857

[bjc12505-bib-0061] Uljarević, M. , Frazier, T. W. , Jo, B. , Scahill, L. , Youngstrom, E. A. , Spackman, E. , Phillips, J. M. , Billingham, W. , & Hardan, A. (2023). Dimensional assessment of restricted and repetitive behaviors: Development and preliminary validation of a new measure. Journal of the American Academy of Child & Adolescent Psychiatry, 62(5), 568–581. 10.1016/j.jaac.2022.07.863 36526162

[bjc12505-bib-0062] Van Buuren, S. , & Groothuis‐Oudshoorn, K. (2011). Mice: Multivariate imputation by chained equations in R. Journal of Statistical Software, 45, 1–67. 10.18637/jss.v045.i03

[bjc12505-bib-0063] Velicer, W. F. , Eaton, C. A. , & Fava, J. L. (2000). Construct explication through factor or component analysis: A review and evaluation of alternative procedures for determining the number of factors or components. In R. D. Goffin & E. Helmes (Eds.), Problems and solutions in human assessment: Honoring Douglas N. Jackson at seventy (pp. 41–71). Springer US. 10.1007/978-1-4615-4397-8_3

[bjc12505-bib-0064] Velicer, W. F. , & Fava, J. L. (1998). Affects of variable and subject sampling on factor pattern recovery. Psychological Methods, 3(2), 231–251. 10.1037/1082-989X.3.2.231

[bjc12505-bib-0065] Wager, T. D. , Jonides, J. , & Reading, S. (2004). Neuroimaging studies of shifting attention: A meta‐analysis. NeuroImage, 22(4), 1679–1693. 10.1016/j.neuroimage.2004.03.052 15275924

[bjc12505-bib-0066] Watkins, M. W. (2018). Exploratory factor analysis: A guide to best practice. Journal of Black Psychology, 44(3), 219–246. 10.1177/0095798418771807

[bjc12505-bib-0067] Wear, H. J. , Wedderburn, C. J. , Mioshi, E. , Williams‐Gray, C. H. , Mason, S. L. , Barker, R. A. , & Hodges, J. R. (2008). The Cambridge behavioural inventory revised. Dementia and Neuropsychologia, 2(2), 102–107. 10.1590/s1980-57642009dn20200005 29213551 PMC5619578

[bjc12505-bib-0068] Wecker, N. S. , Kramer, J. H. , Hallam, B. J. , & Delis, D. C. (2005). Mental flexibility: Age effects on switching. Neuropsychology, 19(3), 345–352. 10.1037/0894-4105.19.3.345 15910120

[bjc12505-bib-0069] Whiting, D. L. , Deane, F. P. , Simpson, G. K. , McLeod, H. J. , & Ciarrochi, J. (2017). Cognitive and psychological flexibility after a traumatic brain injury and the implications for treatment in acceptance‐based therapies: A conceptual review. Neuropsychological Rehabilitation, 27(2), 263–299. 10.1080/09602011.2015.1062115 26156228

[bjc12505-bib-0070] Wilson, C. G. , Nusbaum, A. T. , Whitney, P. , & Hinson, J. M. (2018). Age‐differences in cognitive flexibility when overcoming a preexisting bias through feedback. Journal of Clinical and Experimental Neuropsychology, 40(6), 586–594. 10.1080/13803395.2017.1398311 29161963

