# Supplementary material for: Development of the Flexibility in Daily Life scale to measure multidimensional cognitive and behavioural flexibility in health and disease
Source: Br J Clin Psychol. 2024 Sep 22;64(2):315–29. doi: 10.1111/bjc.12505 (PMC12057328; doi:10.1111/bjc.12505)
Supplement: Supplementary file 1 — Appendix S1. [file BJC-64-315-s001.docx]

**Development of the Flexibility in Daily Life scale to measure multidimensional cognitive and behavioural flexibility in health and disease**

**Supplementary Material**

Files included:

1. **Supplementary Methods: Original 37-item version of the Flexibility in Daily Life (FIDL) scale**
2. **Supplementary Methods: Revised 21-item version of the Flexibility in Daily Life (FIDL) scale**
3. **Supplementary Methods: FIDL Scoring Instructions**

**Supplementary Methods**

**1. Original 37-item version of the Flexibility in Daily Life (FIDL) scale**

We are interested in how often you experience different types of thinking or behaving in your daily life. Using the scale below, please circle ***how often you experience each of the examples***. There is no correct or incorrect answer. Try to think about your experiences over the last 2 weeks.

|  | Never | Rarely | Sometimes | Often | All the time |
| --- | --- | --- | --- | --- | --- |
| 1. I insist on following my specific beliefs and values. | **1** | **2** | **3** | **4** | **5** |
| 1. I insist on eating the same foods or at the same time every day. | **1** | **2** | **3** | **4** | **5** |
| 1. I find it challenging to multitask or to manage multiple demands on my attention. | **1** | **2** | **3** | **4** | **5** |
| 1. I become frustrated or upset if things don't go to plan. | **1** | **2** | **3** | **4** | **5** |
| 1. I can modify my plans when there is a change in circumstances (e.g., change of time or venue, unexpected weather, something is unavailable).* | **1** | **2** | **3** | **4** | **5** |
| 1. I feel anxious or stressed when faced with unfamiliar situations or people. | **1** | **2** | **3** | **4** | **5** |
| 1. I am open to new experiences and trying new things.* | **1** | **2** | **3** | **4** | **5** |
| 1. I tend to have very strong interests, which I insist on pursuing. | **1** | **2** | **3** | **4** | **5** |
| 1. I have specific routines related to my meals/food (e.g. needing to sit in the same spot, use the same cutlery). | **1** | **2** | **3** | **4** | **5** |
| 1. I can easily change my approach to a task if shown a better way.* | **1** | **2** | **3** | **4** | **5** |
| 1. I have a broad range of interests.* | **1** | **2** | **3** | **4** | **5** |
| 1. I often repeat particular words or phrases in exactly the same way (e.g., catchphrases). | **1** | **2** | **3** | **4** | **5** |
| 1. I struggle to get rid of my belongings, even when they have no use (e.g., an old newspaper). | **1** | **2** | **3** | **4** | **5** |
| 1. I like to follow specific routines when I leave the house (e.g., always taking the same route, always being dropped off at the same spot). | **1** | **2** | **3** | **4** | **5** |
| 1. I have to be right, even if it means not listening to others’ opinions. | **1** | **2** | **3** | **4** | **5** |
| 1. I expect others to uphold the same ideas, values, and beliefs as mine. | **1** | **2** | **3** | **4** | **5** |
| 1. I need to repeat some actions/tasks until they feel “just right”. | **1** | **2** | **3** | **4** | **5** |
| 1. I find it hard to let go of my ideas or beliefs, even when presented with new information. | **1** | **2** | **3** | **4** | **5** |
| 1. I feel like I need to repeat certain actions (e.g., pacing, tapping, checking). | **1** | **2** | **3** | **4** | **5** |
| 1. I need to keep objects in my home in a specific place (e.g., ornaments, tools, utensils, appliances). | **1** | **2** | **3** | **4** | **5** |
| 1. I get overly focused on small details even when it’s not helpful to do so. | **1** | **2** | **3** | **4** | **5** |
| 1. I am interested in some object(s) because of their visual properties (e.g., colour, shape, texture). | **1** | **2** | **3** | **4** | **5** |
| 1. I ruminate or brood over the same matters or thoughts. | **1** | **2** | **3** | **4** | **5** |
| 1. I repetitively count and/or order objects in my environment. | **1** | **2** | **3** | **4** | **5** |
| 1. When interrupted, I can easily switch back to what I was previously doing.* | **1** | **2** | **3** | **4** | **5** |
| 1. I prefer to follow a familiar routine or a specific way of doing things. | **1** | **2** | **3** | **4** | **5** |
| 1. I prefer it when people behave in a specific way or according to a set of rules during activities. | **1** | **2** | **3** | **4** | **5** |
| 1. I can easily adapt to changes in the topic of conversation.* | **1** | **2** | **3** | **4** | **5** |
| 1. I find it hard to adjust my behaviour in response to feedback or criticism. | **1** | **2** | **3** | **4** | **5** |
| 1. I have specific interests in items or objects that others find unusual. | **1** | **2** | **3** | **4** | **5** |
| 1. I tend to think in black and white terms (e.g., ‘all-or-nothing', 'good or bad'). | **1** | **2** | **3** | **4** | **5** |
| 1. I can adapt my behaviour according to the social situation (e.g., casual versus formal settings)* | **1** | **2** | **3** | **4** | **5** |
| 1. I prefer to wear the same type of clothes or outfits every day. | **1** | **2** | **3** | **4** | **5** |
| 1. I insist on doing certain things at a specific time every day, regardless of the situation or context. | **1** | **2** | **3** | **4** | **5** |
| 1. I am very unsettled by last-minute changes to my plans. | **1** | **2** | **3** | **4** | **5** |
| 1. I have trouble shifting my attention from one thing to another. | **1** | **2** | **3** | **4** | **5** |
| 1. I have trouble thinking outside the box or coming up with solutions to problems. | **1** | **2** | **3** | **4** | **5** |

**2. Revised 21-item version of the Flexibility in Daily Life (FIDL) scale**

We are interested in how often you experience different types of thinking or behaving in your daily life. Using the scale below, please circle ***how often you experience each of the examples***. There is no correct or incorrect answer. Try to think about your experiences over the last 2 weeks.

|  | Never | Rarely | Sometimes | Often | All the time |
| --- | --- | --- | --- | --- | --- |
| 1. I insist on eating the same foods or at the same time every day. | **1** | **2** | **3** | **4** | **5** |
| 1. I become frustrated or upset if things don't go to plan. | **1** | **2** | **3** | **4** | **5** |
| 1. I can modify my plans when there is a change in circumstances (e.g., change of time or venue, unexpected weather, something is unavailable).* | **1** | **2** | **3** | **4** | **5** |
| 1. I feel anxious or stressed when faced with unfamiliar situations or people. | **1** | **2** | **3** | **4** | **5** |
| 1. I have specific routines related to my meals/food (e.g. needing to sit in the same spot, use the same cutlery). | **1** | **2** | **3** | **4** | **5** |
| 1. I can easily change my approach to a task if shown a better way.* | **1** | **2** | **3** | **4** | **5** |
| 1. I often repeat particular words or phrases in exactly the same way (e.g., catchphrases). | **1** | **2** | **3** | **4** | **5** |
| 1. I like to follow specific routines when I leave the house (e.g., always taking the same route, always being dropped off at the same spot). | **1** | **2** | **3** | **4** | **5** |
| 1. I have to be right, even if it means not listening to others’ opinions. | **1** | **2** | **3** | **4** | **5** |
| 1. I expect others to uphold the same ideas, values, and beliefs as mine. | **1** | **2** | **3** | **4** | **5** |
| 1. I need to repeat some actions/tasks until they feel “just right”. | **1** | **2** | **3** | **4** | **5** |
| 1. I find it hard to let go of my ideas or beliefs, even when presented with new information. | **1** | **2** | **3** | **4** | **5** |
| 1. I feel like I need to repeat certain actions (e.g., pacing, tapping, checking). | **1** | **2** | **3** | **4** | **5** |
| 1. I get overly focused on small details even when it’s not helpful to do so. | **1** | **2** | **3** | **4** | **5** |
| 1. I repetitively count and/or order objects in my environment. | **1** | **2** | **3** | **4** | **5** |
| 1. When interrupted, I can easily switch back to what I was previously doing.* | **1** | **2** | **3** | **4** | **5** |
| 1. I prefer it when people behave in a specific way or according to a set of rules during activities. | **1** | **2** | **3** | **4** | **5** |
| 1. I can easily adapt to changes in the topic of conversation.* | **1** | **2** | **3** | **4** | **5** |
| 1. I have specific interests in items or objects that others find unusual. | **1** | **2** | **3** | **4** | **5** |
| 1. I can adapt my behaviour according to the social situation (e.g., casual versus formal settings)* | **1** | **2** | **3** | **4** | **5** |
| 1. I am very unsettled by last-minute changes to my plans. | **1** | **2** | **3** | **4** | **5** |

**3. FIDL Scoring Instructions**

| **Scale** | **Items** | **Score** |
| --- | --- | --- |
| *Repetition* | 7, 11, 13, 14, 15, 19 |  |
| *Switching* | 3*, 6*, 16*, 18*, 20* |  |
| *Predictability/Control* | 2, 4, 17, 21 |  |
| *Routine* | 1, 5, 8 |  |
| *Thoughts/Beliefs* | 9, 10, 12 |  |
| ***Total FIDL*** | Sum of all sub-scales |  |

*reverse-scored item
